# Inertial flywheel knee- and hip-dominant hamstring strength exercises in professional soccer players: Muscle use and velocity-based (mechanical) eccentric overload

**DOI:** 10.1371/journal.pone.0239977

**Published:** 2020-10-02

**Authors:** Luis Suarez-Arrones, F. Javier Núñez, Pilar Lara-Lopez, Valter Di Salvo, Alberto Méndez-Villanueva

**Affiliations:** 1 Department of Sport and Informatics, Section of Physical Education and Sport, Pablo de Olavide University, Sevilla, Spain; 2 Performance Department, FC Basel, Basel, Switzerland; 3 Football Performance & Science Department, ASPIRE Academy, Doha, Qatar; 4 Department of Movement, Human and Health Sciences, University of Rome “Foro Italico”, Rome, Italy; 5 Qatar Football Association, Doha, Qatar; Universidade Federal de Mato Grosso do Sul, BRAZIL

## Abstract

The primary aim of the present study was to analyze mechanical responses during inertial knee- and hip-dominant hamstring strengthening exercises (flywheel leg-curl and hip-extension in conic-pulley), and the secondary aim was to measure and compare regional muscle use using functional magnetic resonance imaging. Mean power, peak power, mean velocity, peak velocity and time in the concentric (CON) and eccentric (ECC) phases were measured. The transverse relaxation time (T2) shift from pre- to post-exercise were calculated for the biceps femoris long (BFl) and short (BFs) heads, semitendinosus (ST) and semimembranosus (SM) muscles at proximal, middle and distal areas of the muscle length. Peak and mean power in flywheel leg-curl were higher during the CON than the ECC phase (p<0.01). ECC peak power was higher than CON phase (p<0.01) in conic-pulley hip-extension exercise, while mean power was higher during the CON than ECC phase (p<0.01). Flywheel leg-curl showed a higher T2 values in ST and BFs and BFl (p<0.05), while the conic-pulley hip-extension had a higher T2 values in the proximal region of the ST and BFl (p<0.05). In conclusion, ECC overload was only observed in peak power during the conic-pulley hip-extension exercise. Flywheel leg-curl involved a greater overall use of the 4 muscle bellies, more specifically in the ST and BFs, with a selective augmented activity (compared with the conic-pulley) in the 3 regions of the BFs, while conic-pulley hip-extension exercise selectively targeted the proximal and medial regions of the BFl. Physiotherapists and strength and conditioning coaches should consider this when optimizing the training and recovery process for hamstring muscles, especially after injury.

## Introduction

Hamstring injuries are the most common soccer-related muscle injury [1,2] representing more than 37% of all soccer-related injuries and requiring long rehabilitation periods [1,2]. The hamstring muscles comprise the biceps femoris muscle [long head (BFl) and short head (BFs)], semitendinosus muscle (ST) and semimembranosus muscle (SM) located in the posterior of the thigh. The BF is the most commonly strained muscle in soccer players [2,3], accounting for 53–85% of the total injuries in the hamstring complex [2,4,5], whereas the ST and SM muscles are less often injured [2,6,7]. The hamstring muscles also act as an anterior cruciate ligament (ACL) synergist, decreasing the anterior translation of the tibia and reducing internal tibial rotation [8]. Previous studies showed that the ST muscle could play a key role as the most important neuromuscular ACL agonist, suggesting that an increasing in ST muscle activity via neuromuscular training intervention can be a key part of ACL preventive strategy [9]. All this information helps to understand the importance of this muscle group for performance and from the point of view of both prevention of and recovery from injuries. Accordingly, most preventive strategies aimed at decreasing hamstring and non-contact ACL injuries include some sort of neuromuscular training of the hamstring muscles as an essential aspect [10–13].

The hamstring muscles are involved in knee flexion and/or hip extension, and as previously reported in previous studies, BFl is selectively recruited during hip extension exercises [7,14], whereas ST and BFs are activated in knee-flexion exercises [14–16]. Weakness in eccentric knee flexor strength has been proposed as one of the main risk factors for hamstring injury [17,18], although a recent study showed that Nordic hamstring strength was not associated with increased or decreased risk of sustaining a hamstring muscle injury [19]. Using an isokinetic test, lower hamstring eccentric strength adjusted for body weight was identified as a (weak) risk factor associated with hamstring injury in professional soccer players [20]. In addition to this, strength training has been proposed as a preventive measure in order to reduce hamstring muscle injuries and thus medical costs in soccer [10–12]. Mechanical eccentric overload is suggested to achieve muscle hypertrophy, strengthening of the musculotendinous tissue and protection of the muscle-tendon complex against further injury [21–24]. Currently, there are devices that use inertia to provide a source of linear resistance independent of gravity, using a tether wrapped around a horizontal cylinder-shaped shaft (i.e., flywheel leg curl) [25] or a vertical cone-shaped shaft (i.e., Versa-pulley) [26] producing a resistance during the entire range of motion [23]. The inertial torque is a function of the mass of the disc, the disc’s radius of gyration and the disc’s angular acceleration [27], which together allow these devices to offer eccentric overload [25]. The kinetic energy increases based on rotational speed and once the concentric phase was finalized, the cord rewinds and the trainee must resist the pull of the inertial device executing a braking causing this an eccentric muscle action [23]. The magnitude of this overload is largely dependent on previous experience with this technology as was shown by Tous-Fajardo et al. [25], and the experience with these devices is crucial to optimize training. Previous studies using flywheel technology have shown benefits such us improved performance [24,28,29], chronic adaptations on muscular strength, power and gains in lean mass [28–30], higher force and power production combined with low energy expenditure [28] or reduced hamstring injuries in professional soccer players [11,22,28]. It should be noted that with the use of this flywheel technology a familiarization process is required and there is a lack of standard procedures for exercise loading prescription and limited evidence with elite athletes [28]. In addition, limited information exists describing the basic kinematics of these devices as used during hip extension and knee flexion exercises. Tour-Fajardo et al. [25] reported kinematic data measuring the velocity with a lineal encoder and force with a strain gauge during all-out knee flexions in a flywheel leg-curl, showing that this flywheel device offered eccentric overload to experienced athletes in peak force or velocity, but not in mean force and mean velocity. In the same line, Núñez et al. [31] did not obtain any force or velocity eccentric overload during the squat exercise using two different inertial devices (conic-pulley and Yo-Yo flywheel). When compared with free weights, Núñez et al. [32] reported, in experienced rugby players, a substantially greater eccentric overload and augmented metabolic demands with these devices [32]. Thus, despite that it is likely that inertial devices can generate augmented eccentric overloads compared with free weights and other traditional devices [32], it is not clear what magnitude of eccentric overload can be generated in relation to the concentric phase with these devices [25,31].

Functional magnetic resonance imaging (fMRI) is used to display the physiological changes that occur in muscles activated during exercise, providing detailed anatomical analysis of associated soft issues, which is lacking in electromyography experiments [15,16,33,34]. Previous studies have reported that changes in response to resistance training occur non-uniformly along the length of the muscle [35,36]. These changes have been attributed to region-specific muscle activation, as assessed via the transverse relaxation time (T2) (a quantitative measurement of muscle activity), using fMRI before and immediately following the exercise [14,37]. To date, only few studies have investigated individual muscle use, by means of fMRI, of different hamstring resistance exercises in professional soccer players [14,37]. Those previous studies showed that different hamstring muscles and specific-regions within each muscle are likely to be selectively activated during different resistance exercises [14,37], although none of them have described the basic kinematics of these devices during the CON and ECC phases. To our knowledge, there is no information describing both the mechanical responses and region-specific muscle recruitment after concentric–eccentric training with flywheel devices during knee- and hip-dominant (i.e. strengthening exercises). Therefore, the primary aim of the present study was to analyze mechanical responses during inertial knee- and hip-dominant hamstring strengthening exercises (Conic-Pulley and Yo-Yo flywheel), and the secondary aim was to measure and compare regional muscle use using fMRI.

## Methods

### Participants

The study examined 19 male elite professional soccer players (age 20.4 ± 3.9 years; height 180.0 ± 3.0; weight 72.3 ± 7.5 kg). Players belonged to the reserve squad of a Spanish La Liga club that competed in the UEFA Champions League, and participated in ~ 8 hours of soccer training plus 1 or 2 competitive games per week. All the team usually supplemented the soccer training with a basic strength-training program combining free weights, Russian Belt and Nordic hamstring exercise, but without experience with inertial devices. Players were randomly assigned to one of the two groups/exercises: 10 players performed flywheel leg-curl (LC) exercises and 9 players used hip-extension Versa Pulley (VP) exercises. The inclusion criterion was to be available to play an official game with the squad, and the testing procedure was conducted during preseason. There was an exclusion because one player couldn´t complete the exercise protocol. Thus, of the 19 initially examined players, 18 players remained for statistical analysis (LC, n = 10; VP, n = 8). The purpose and experimental protocol was explained to the players and written informed consent was obtained from the players (or tutor for players under 18). The Anti-Doping Lab Institutional Review Board (Qatar) conformed to the recommendations of the Declaration of Helsinki, approved the present study.

### Experimental design

To investigate the mechanical variables and regional-specific differences of fMRI muscle measurements in two hamstring exercises, the present study used a repeated-measures research design before and after completing an acute strength training session with an LC or VP.

### Procedures

On the experiment day the players underwent fMRI of both thighs at rest, 30 min before beginning the training session. Before the strength training protocol, players performed a 15-min standardized warm up that included jogging, lower limb joint mobility exercises, dynamic and active stretching exercises, running technique drills, bodyweight squat and frontal lunge exercises, and one submaximal set of 8 repetitions of LC or VP. After completing the strength training protocol the players underwent fMRI of both thighs (within 3 min), and each player’s session RPE was collected using the Borg scale-10 (30 min after the session finished) [38].

### Exercise protocol

The training session consisted of 4 sets of 8 repetitions of one of the two exercises (i.e. LC or VP) and non a priori power calculation was done. The smallest possible resistance was employed (0.07208 kg/m^2^ moment inertia for LC and 0.21964 kg/m^2^ moment inertia for VP). Most of the players only experienced two familiarization submaximal sets of 6–8 repetitions with this technology 2–3 days before the experiment day. There was a 2-min rest between each set, during which the subjects rested in a standing position. All the repetitions performed by each player were recorder for further analysis. Mean power, peak power, mean velocity, peak velocity and time during concentric and eccentric phase was sampled at 100 Hz using a rotatory encoder (SmartCoach^™^, SmartCoach Europe AB, Stockholm, Sweden) and associated software (SmartCoach^®^ v.5.2.0.5). The concentric: eccentric ratio was calculated for mean power (*P*_*M*_ *CON*: *ECC ratio)*, peak power (*P*_*p*_ *CON*: *ECC ratio)* and time (*Time CON*:*ECC ratio)*. Internal training load was quantified by analyzing the RPE of each training session. The RPE value was multiplied by the number of repetitions [39,40] to estimate the RPE-derived internal training load (sRPE-TL).

#### Flywheel leg-curl

**Fig 1:**
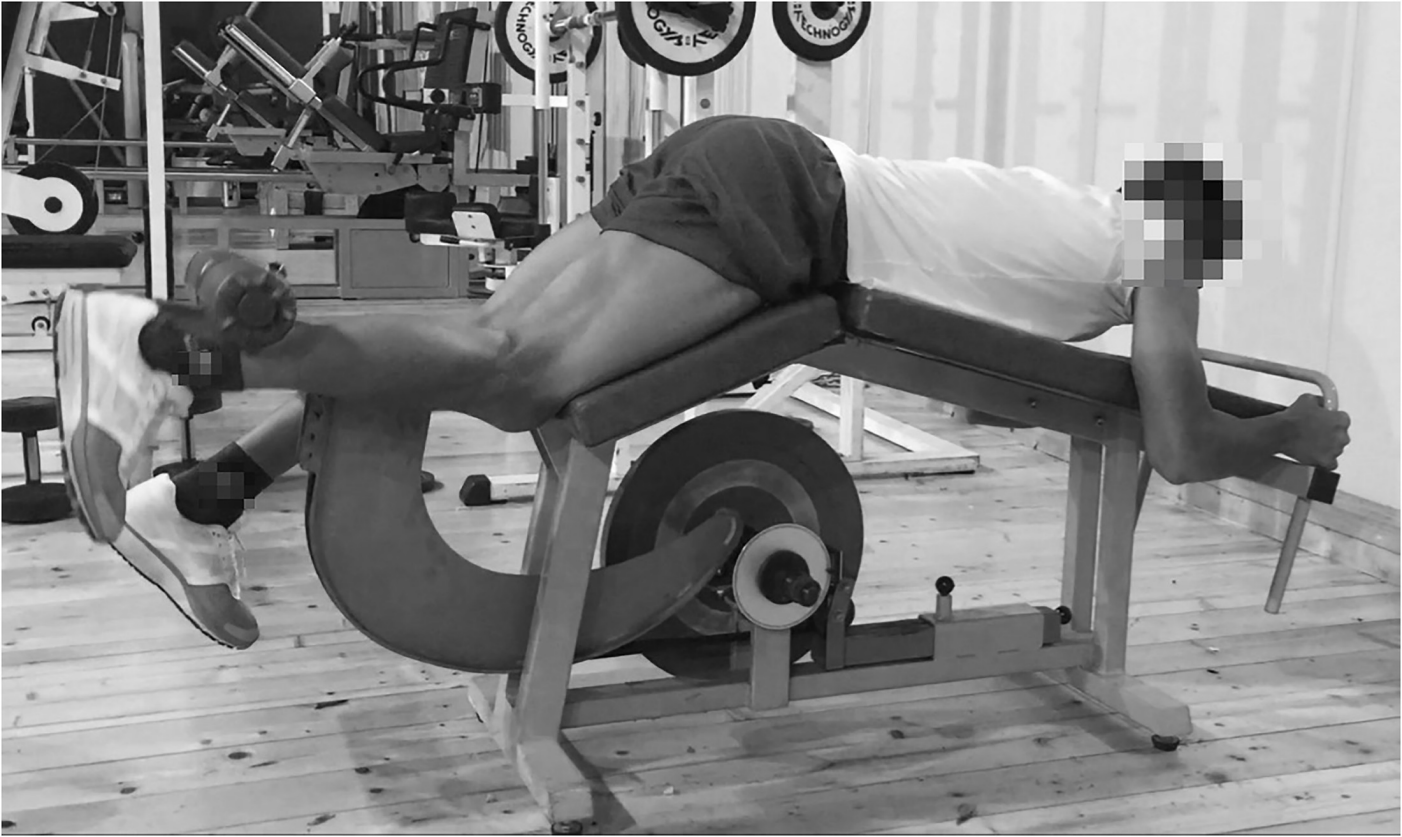
Flywheel leg-curl.

A non-gravity device that provided a source of linear resistance (0.07208 kg/m^2^ moment of inertia) from a tether wrapped around a horizontal cylinder-shaped shaft was used (LC: Leg curl YoYo Technology AB, Stockholm, Sweden) (Fig 1). Players were in a supine head-down position and performed unilateral knee flexion with the dominant leg (the hip was fixed at a 140° angle and the contra-lateral leg rested firmly on the floor), applying force at maximal velocity during the acceleration phase of the movement (CON: Concentric), and attempting to stop the movement at the end of the deceleration phase (ECC: Eccentric) without reaching full extension [11,25].

#### Hip-extension versa-pulley

**Fig 2:**
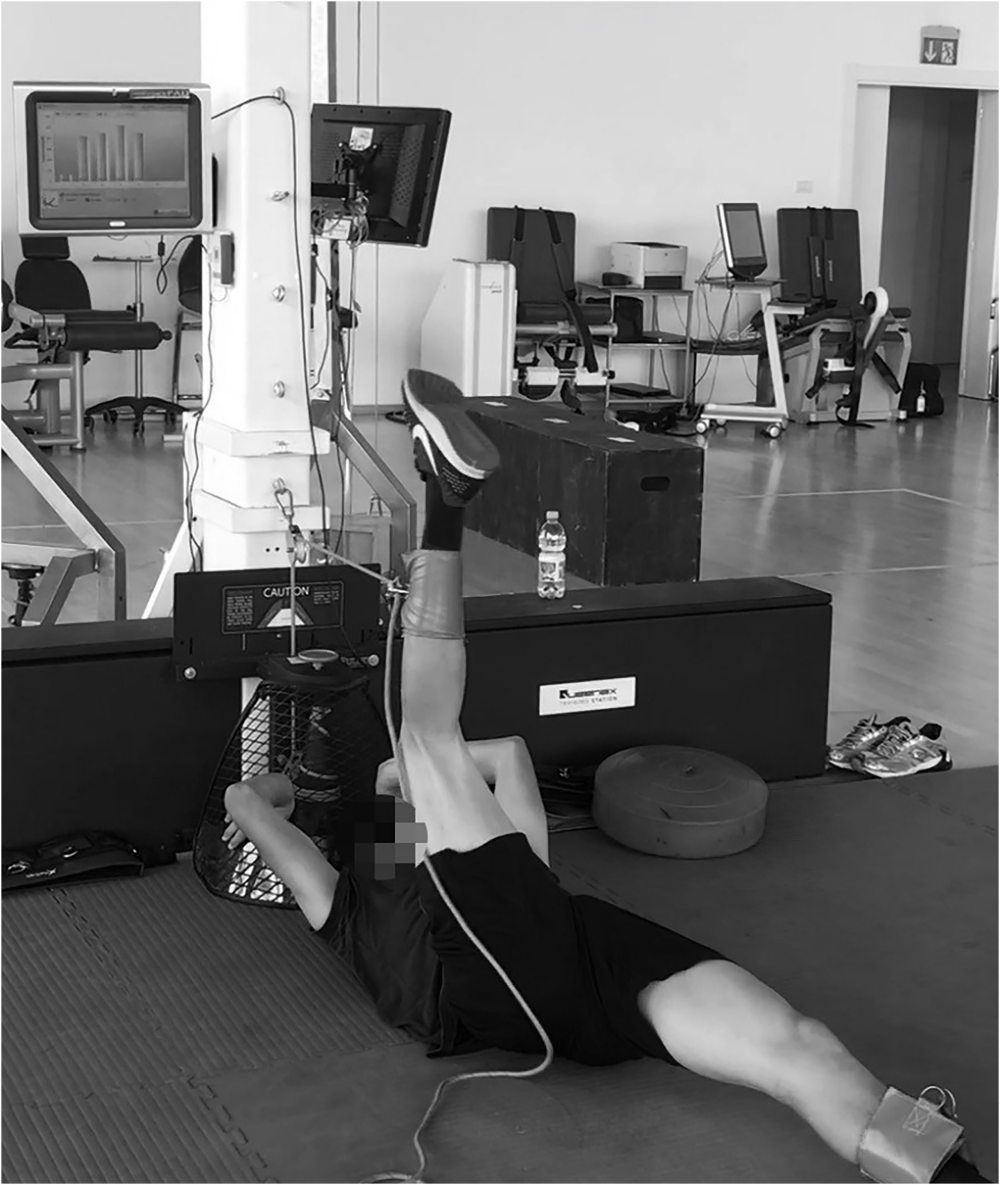
Hip extension in versa pulley.

A non-gravity device that provided a source of linear resistance (0.21964 kg/m^2^ moment of inertia) from a tether wrapped around a vertical cone-shaped shaft was used (VP: (VersaPulley portable; VersaClimber, Halesowen, UK) (Fig 2). Players were in a supine head-up position, with the strap placed around the ankle, and performed a hip extension with the dominant leg (with a slight knee extension, while the non-dominant leg was blocked by a coach to avoid movement) applying force at maximal velocity during CON, and attempting to stop the movement at the end of ECC.

### Imagine technique

All fMRI measurements were obtained using a 3 T whole-body imager with surface phased-array coils (Siemens, Erlagen, Germany). For the fMRI scans, the subjects were in a supine position with their knees extended. Once the subject was positioned inside the MRI chamber, the thighs of both legs were kept parallel to the fMRI table, and a custom-made foot-restraint device was used to standardize and fix limb position and avoid any compression of the thigh muscles. Subjects were supine on the MR gurney with the thighs covered with one 32- and 2 flexible 4-channel coils, respectively in the proximal and distal segments. Twelve cross-sectional images of the thighs of both legs were obtained, starting at the very distal margin of the ischial tuberosity, and using the following scan sequences: (a) axial fat-suppressed proton density, TR 3000 ms, TE 30–33, eco train 4, slice thickness 3.5 mm, gap 28 mm, FOV 400 × 290 mm, matrix 320 × 180 and ipat 2; (b) axial T2 mapping, TR 1000 ms, TE (18, 36, 54, 72, 90, 108), eco train 6, FOV 400 × 400 mm, matrix 256 × 256, slice thickness 3.5 mm and gap 28 mm. A parametric image was generated from a T2 mapping sequence using the Leonardo workstation (Siemens). Scout images and anatomical landmarks were obtained to ensure identical and time-efficient positioning in pre- and post- scans.

T2 values of BFl, BFs, SM, and ST muscles from the dominant leg were calculated using eFilm Lite v.3.1 software (Merge Healthcare, Chicago, IL). Using the fat-suppressed images to detect any confounding artifacts (e.g., vessels, fat), a circular region of interest (ROI, mm) was selected for individual muscles (BFl, BFs, SM, and ST) in each of the T2 mapping images where muscles were visible. Following the pre-exercise scan analysis, the same sized circular ROIs were placed in the T2 images of the post-exercise scan, to ensure identical positioning as in the pre-exercise analysis. Site-specific muscle use was calculated after each exercise by obtaining the baseline and post-exercise average values of the first 30% axial scans, where each muscle was visible starting from the hip/knee joint (proximal and distal portions, respectively), and the middle scans (from 30% to 70%; mid portion) [16]. Two researchers, blinded to the origin of all images, independently analyzed all images. The intraclass correlation coefficients and coefficients of variation for the inter-rater agreement of the T2 values for the different muscle were: BFl (0.95, 2.5%), BFs (0.98, 1.8%, 0.77), ST (0.97, 1.9%) and SM (0.89, 3.8%).

The following variables were analysed:

1. T2 changes within and between muscle bellies, representing the amount of metabolic muscle activity between the scans (T2 change = T2post-T2pre / T2 pre) [41].
2. The proportional shares of the different hamstring muscle (BFl, BFs, SM and ST) regions (proximal, medial and distal) within the entire hamstring T2 value change, for which the T2 change in each muscle belly region was normalized to the summated changes of all muscle belly regions [proportional activity = T2 change (BFl region or BFs region or SM region or ST region)/T2 change (T2 changes of each region of BFl+BFs+SM+ST)] [41].

### Statistical analysis

Data are presented as mean ± standard deviation (SD) and coefficient of variation (CV) [(SD/mean x 100)]. Descriptive statistics were calculated on each variable and Shapiro-Wilk test were used to verify normality (SPSS 2018, Inc., Chicago, IL). A one-way analysis of variance (ANOVA) was used to determine differences between groups and Bonferroni’s post-hoc tests were used to identify any localized effects. Differences within group were determined using Student’s dependent *t*-test. Statistical significance was set at p<0.05 (95%CI). The standardized difference or effect size (ES, 95% confidence interval [95%CI]) in the selected variables was calculated. Threshold values for assessing magnitudes of the ES (changes as a fraction or multiple of baseline standard deviation) were >0.20, 0.20, 0.60, 1.2 and 2.0 for trivial, small, moderate, large and very large respectively [42]. To assess the intra-set reliability of the measures, both the intra-class coefficient correlation (ICC) and the coefficient of variation (CV) were used. Pearson’s correlation coefficients were calculated to establish the respective relationships between mechanical variables measured and changes in T2 values of BFl, BFs, SM, and ST muscles.

## Results

### Mechanical variables

Intra-set reliability for hip-extension versa-pulley showed good values for mean power (ICC: 0.99 (0.95; 1.00); CV: 2.6% (1.7; 5.4)), peak power (ICC: 0.95 (0.76; 0.99); CV: 3.8% (2.5; 7.9)), P_M_ CON: ECC ratio (ICC: 0.96 (0.81; 0.99); CV: 3.1% (2.0; 6.4)) and CON: ECC ratio (ICC: 0.95 (0.77; 0.99); CV: 4.2% (2.8; 8.7)). Intra-set reliability for flywheel leg-curl showed good values for mean power (ICC: 1.00 (1.00; 1.00); CV: 2.8% (1.9; 5.9)), peak power (ICC: 0.99 (0.97; 1.00); CV: 4.5% (3.0; 9.4)), P_M_ CON: ECC ratio (ICC: 0.92 (0.65; 0.98); CV: 5.8% (3.8; 12.2)) and CON: ECC ratio (ICC: 0.81 (0.32; 0.96); CV: 16.4% (10.6; 36.2)).

**Fig 3:**
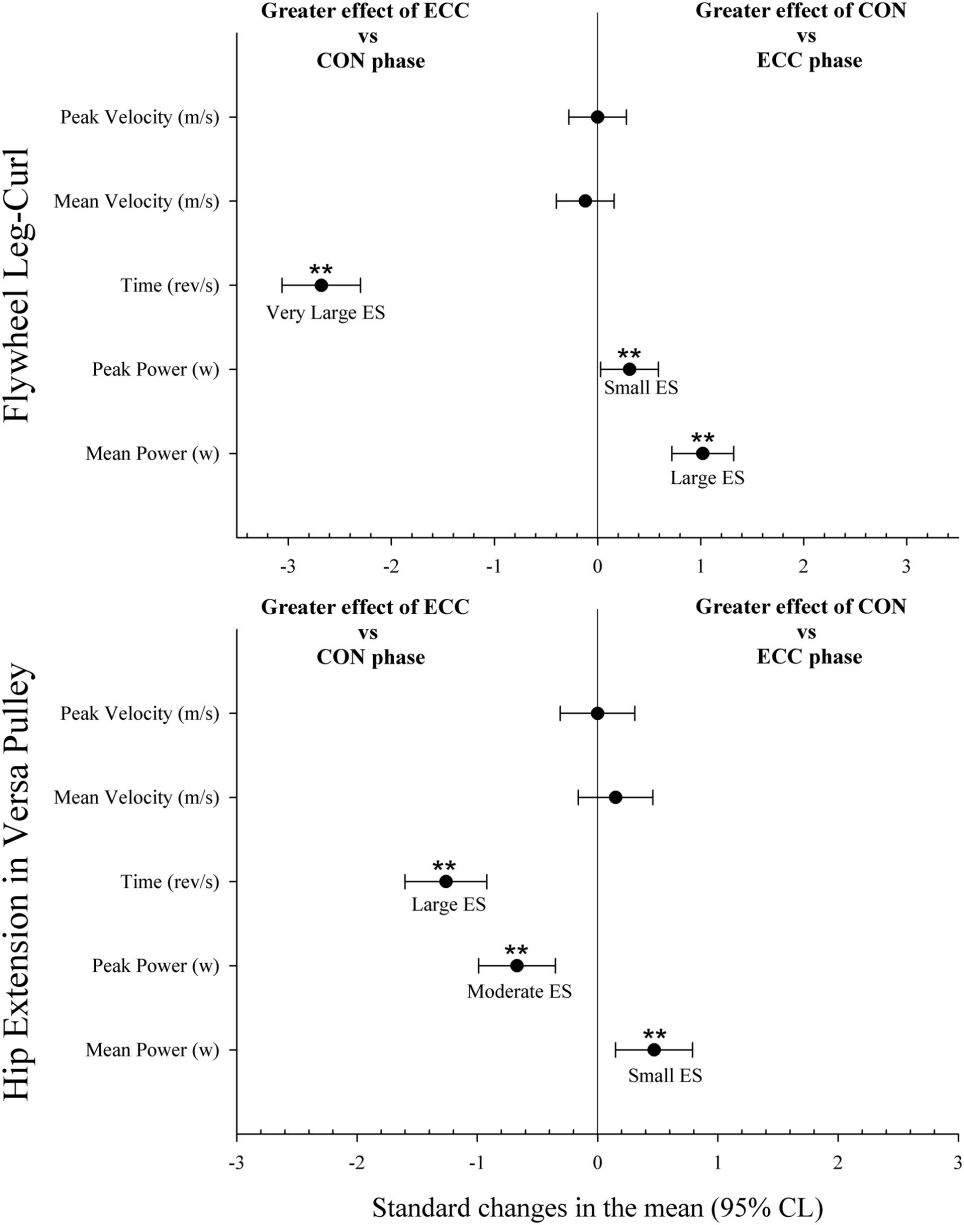
Comparisons between concentric (CON) and eccentric (ECC) phases during flywheel leg-curl and versa-pulley hip-extension exercises.

Mechanical variables during the 4 sets with LC exercise are shown in Table 1. ECC time during the first set was lower than during any other set (p<0.05, ES from 0.33 to 0.55). Comparisons between CON and ECC phases are shown in Fig 3. ECC time was higher than CON time (p<0.01), while peak and mean power were higher during the CON than the ECC phase (p<0.01).

**Table 1:** Mechanical variables during the 4 sets with flywheel leg-curl. Values are mean ± SD.

*Variables*	Sets			
	1	2	3	4
CON mean Power (w)	64.0 ± 22.5	59.4 ± 22.0	59.6 ± 20.2	63.5 ± 22.2
ECC mean Power (w)	45.6 ± 16.9	41.0 ± 16.7	39.6 ± 14.6	41.6 ± 16.5
*P*_*M*_ *CON*: *ECC ratio*	1: 0.71 ± 0.10*	1: 0.68 ± 0.09^d^	1: 0.65 ± 0.11	1: 0.65 ± 0.10
CON peak Power (w)	104.2 ± 32.9	99.8 ± 36.6	100.6 ± 34.5	107.1 ± 36.6
ECC peak Power (w)	99.4 ± 41.7^c^	92.0 ± 41.1	83.5 ± 32.3	90.6 ± 38.4
*P*_*P*_ *CON*: *ECC ratio*	1: 0.94 ± 0.21^c^^,^^d^	1: 0.91 ± 0.20^c^^,^^d^	1: 0.83 ± 0.19	1: 0.83 ± 0.20
CON time (rev/s)	1.20 ± 0.13^d^	1.20 ± 0.14	1.17 ± 0.12	1.14 ± 0.13
ECC time (rev/s)	1.70 ± 0.24*	1.79 ± 0.32	1.86 ± 0.45	1.82 ± 0.33
*Time CON*: *ECC ratio*	1: 1.42 ± 0.14*	1: 1.50 ± 0.27^c^^,^^d^	1: 1.59 ± 0.38	1: 1.61 ± 0.38
CON mean Velocity (m/s)	5.15 ± 0.79	4.96 ± 0.89	4.92 ± 0.81	5.08 ± 0.87
ECC mean Velocity (m/s)	5.20 ± 0.92	5.02 ± 0.93	4.88 ± 0.86	5.11 ± 0.90
CON peak Velocity (m/s)	7.28 ± 1.07	6.98 ± 1.16	6.94 ± 1.13	7.08 ± 1.16
ECC peak Velocity (m/s)	7.28 ± 1.07	6.97 ± 1.16	6.94 ± 1.13	7.08 ± 1.15

CON: Concentric Phase; ECC: Eccentric Phase; P_M_; Mean power; P_P_; Peak power; Rev/s: Revolutions per second

^c^ = significantly different vs 3^rd^ set (p<0.05).

^d^ = significantly different vs 4^rd^ set (p<0.05).

* = significantly different vs others (p<0.05).

Mechanical variables during the 4 sets with VP exercise are shown in Table 2. CON and ECC mean power during the first set was lower than in any other set (p<0.05, ES from 0.36 to 0.56). CON peak power during the first set was lower than in any other set (p<0.05, ES from 0.42 to 0.78), and ECC peak power during the fourth set was higher than any other set (p<0.05, ES from 0.41 to 0.67). CON and ECC time during the first set was higher than during any other set (p<0.05, ES from 0.36 to 0.61). Comparisons between the CON and ECC phases are shown in Fig 3. ECC time and ECC peak power were higher than CON phase (p<0.01), while mean power was higher during the CON than ECC phase (p<0.01).

**Table 2:** Mechanical variables during the 4 sets with hip-extension versa pulley. Values are mean ± SD.

*Variables*	Sets			
	1	2	3	4
CON mean Power (w)	476.7 ± 109.9*	519.1 ± 135.0	512.8 ± 127.3	538.9 ± 126.7
ECC mean Power (w)	410.8 ± 135.3*	453.9 ± 150.5	448.2 ± 142.8	481.9 ± 145.6
*P*_*M*_ *CON*: *ECC ratio*	1: 0.85 ± 0.15^c^^,^^d^	1: 0.86 ± 0.11	1: 0.87 ± 0.11	1: 0.89 ± 0.12
CON peak Power (w)	770.8 ± 119.0*	816.5 ± 166.8^d^	813.8 ± 152.2^d^	870.8 ± 151.9
ECC peak Power (w)	881.9 ± 193.5^c^	916.5 ± 205.1	943.3 ± 242.8	1037.8 ± 221.0*
*P*_*P*_ *CON*: *ECC ratio*	1: 1.16 ± 0.28	1: 1.15 ± 0.30	1: 1.18 ± 0.30	1: 1.22 ± 0.32
CON time (rev/s)	0.83 ± 0.05*	0.81 ± 0.06	0.80 ± 0.06	0.80 ± 0.05
ECC time (rev/s)	1.02 ± 0.23*	0.96 ± 0.13	0.94 ± 0.11	0.92 ± 0.13
*Time CON*: *ECC ratio*	1: 1.23 ± 0.29^c^^,^^d^	1: 1.18 ± 0.16	1: 1.18 ± 0.16	1: 1.15 ± 0.19
CON mean Velocity (m/s)	4.96 ± 0.53*	5.13 ± 0.51	5.11 ± 0.51	5.25 ± 0.53
ECC mean Velocity (m/s)	4.64 ± 0.89*	5.05 ± 0.68	5.10 ± 0.60	5.20 ± 0.73
CON peak Velocity (m/s)	7.53 ± 0.77*	7.74 ± 0.87	7.66 ± 0.83	7.86 ± 0.81
ECC peak Velocity (m/s)	7.53 ± 0.76*	7.74 ± 0.87	7.66 ± 0.83	7.85 ± 0.81

CON: Concentric Phase; ECC: Eccentric Phase; P_M_; Mean power; P_P_; Peak power; Rev/s: Revolutions per second

^c^ = significantly different vs 3^rd^ set (p<0.05).

^d^ = significantly different vs 4^rd^ set (p<0.05).

* = significantly different vs others (p<0.05).

**Fig 4:**
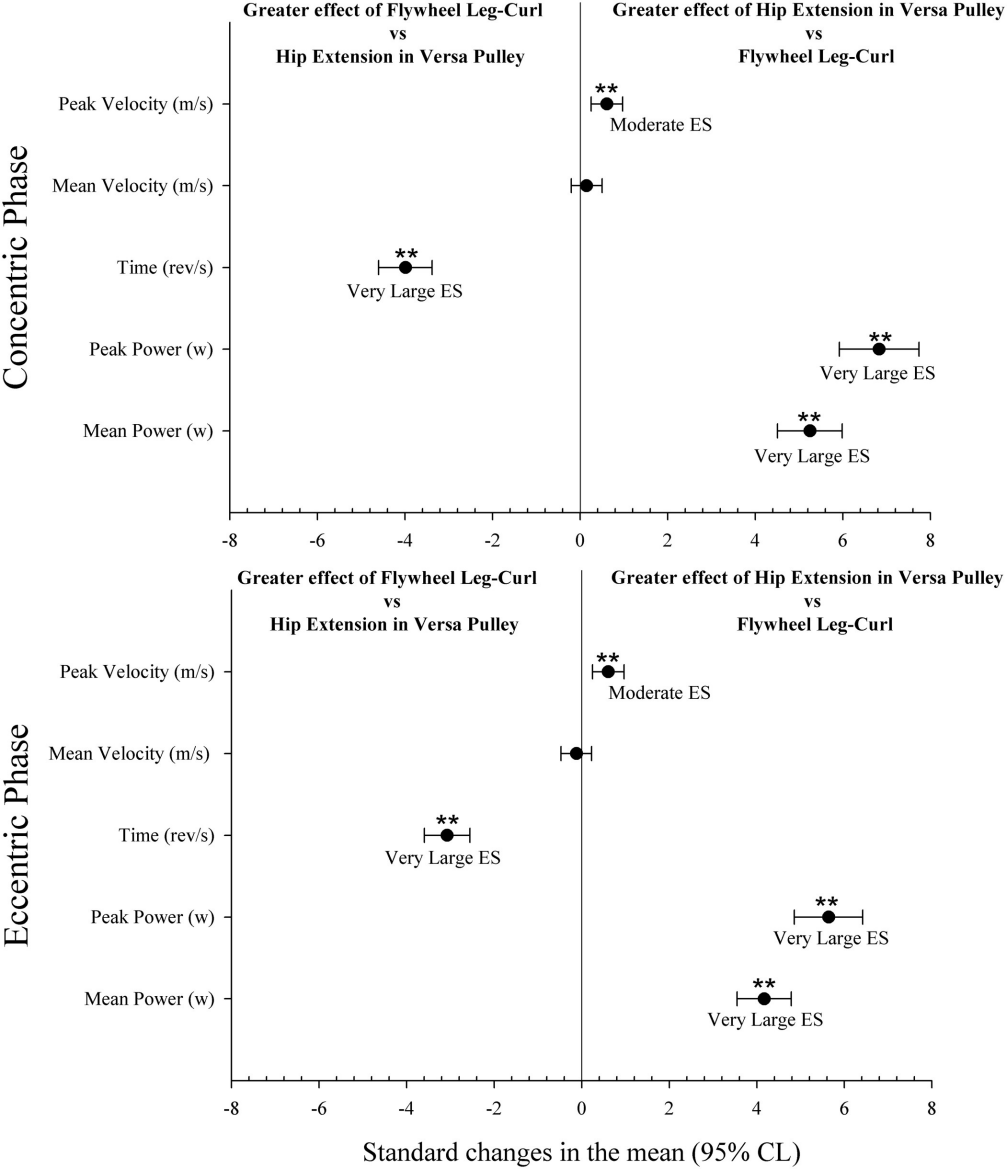
Comparisons between flywheel leg-curl and versa-pulley hip-extension exercises during concentric and eccentric phases.

Comparisons between LC and VP exercises during CON and ECC phases are shown in Fig 4. Time during the CON and ECC phases of LC was higher than for VP (p<0.01). Peak velocity, peak power and mean power during the CON and ECC phases of VP were higher than those in LC (p<0.01).

sRPE-TL was significantly higher after LC exercise (217.6 ± 14.3 AU) than after VP (144.0 ± 18.5 AU) (p<0.01).

### MRI

**Fig 5:**
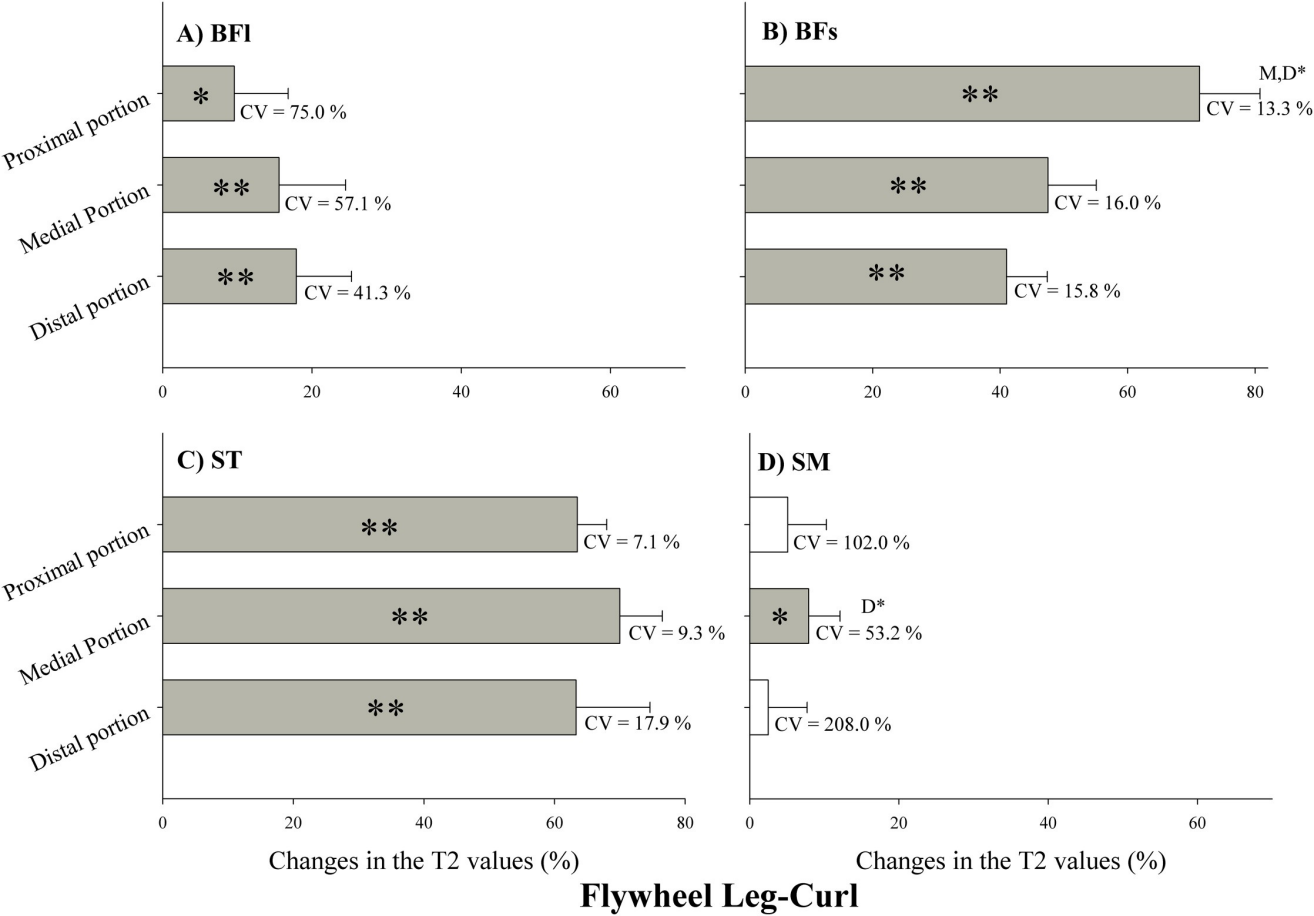
Changes in the transverse relaxation time (T2) values immediately after flywheel leg-curl exercise. Values are given as a percentage of the pre-values. BFl: biceps femoris long head; BFs: Biceps femoris short head; ST: semitendinosus; SM: semimembranosus. CV: coefficient of variation. P: proximal portion; M: medial portion; D: distal portion. ** Significant difference between muscle regions (p<0.01). * Significant difference between muscle regions (p<0.05). Open bars represent no statistical changes.

Changes in T2 values after LC exercise are presented in Fig 5. T2 values were significantly increased in the proximal, medial and distal portion of the BFl (Fig 5A), BFs (Fig 5B) and ST (Fig 5C) (p<0.05), while T2 values were significantly increased only in the medial portion of the SM (Fig 5D) (p<0.05). Changes in the proximal portion of BFs were significantly higher than the changes in the medial and distal portions (Fig 5B) (p<0.01), and changes in the medial portion of SM were significantly higher than the changes in the distal portion (Fig 5D) (p<0.01).

**Fig 6:**
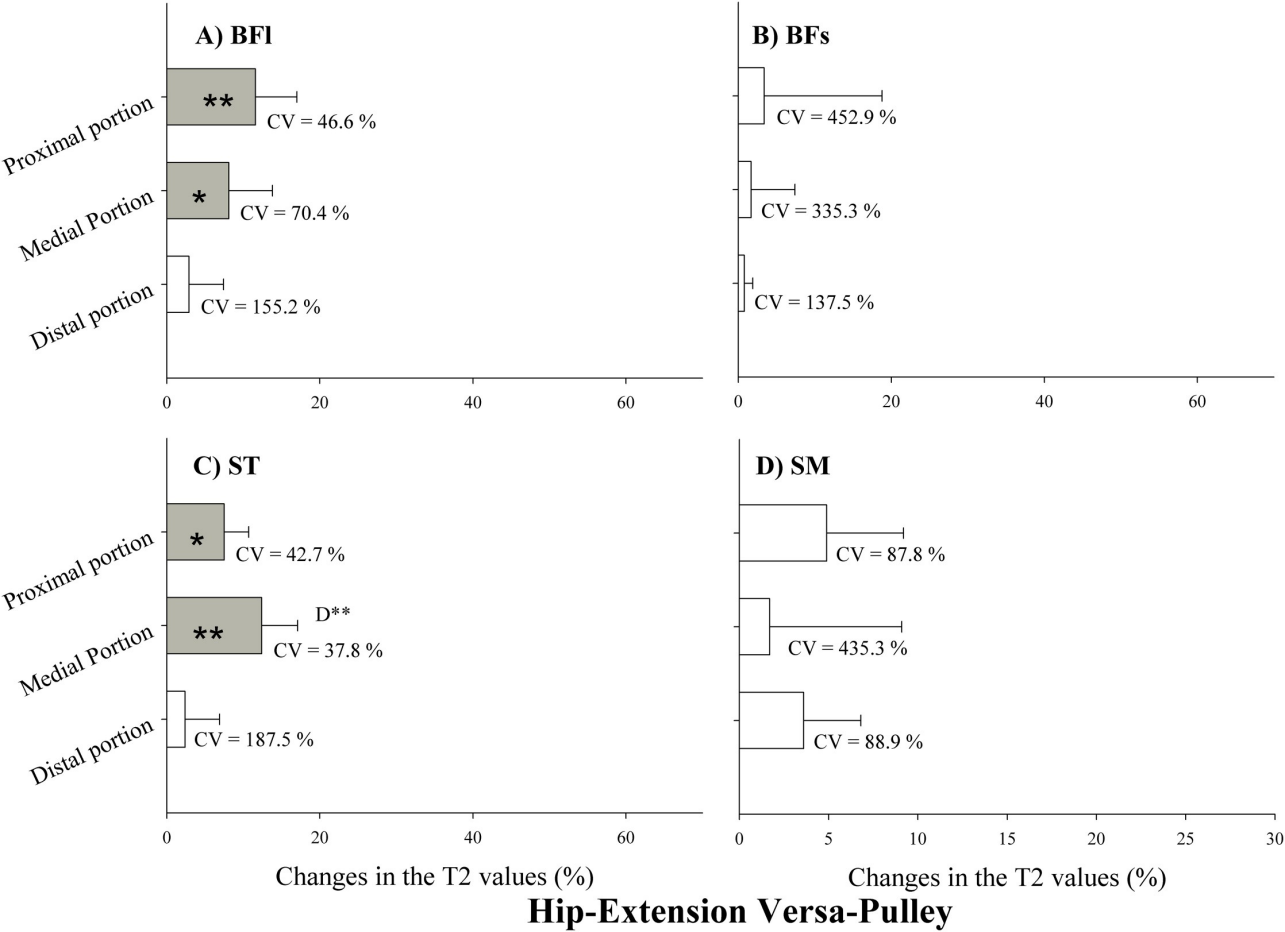
Changes in the transverse relaxation time (T2) values immediately after versa-pulley hip-extension exercise. Values are given as a percentage of the pre-values. BFl: biceps femoris long head; BFs: Biceps femoris short head; ST: semitendinosus; SM: semimembranosus. CV: coefficient of variation. P: proximal portion; M: medial portion; D: distal portion. ** Significant difference between muscle regions (p<0.01). * Significant difference between muscle regions (p<0.05). Open bars represent no statistical changes.

Changes in T2 values after VP exercise are presented in Fig 6. T2 values were significantly increased only in the proximal and medial portions of the BFl and ST (Fig 6A and 6C, respectively) (p<0.05). There were no changes in BFs and SM. Changes in the medial portion of ST were significantly higher than the other changes (Fig 6C) (p<0.01).

The individual contributions of each region of the 4 different muscle bellies to the exercise-related T2 changes are presented in Table 3. The proximal and medial portions of the BFl showed a significant increased use during the VP exercise compared with the LC (p<0.01), while the three regions of the BFs displayed selective use during the LC compared with the VP (p<0.01).

**Table 3:** Comparison between individual share of each hamstring muscle portion (i.e., proximal, medial and distal) in the total T2 shift for the entire hamstring group (portion of 100%) immediately after the flywheel leg-curl and versa-pulley hip-extension exercises.

	Flywheel-Leg Curl (FLC)	Pulley-Hip Extension (PLE)	Standardized Differences (±95%CL)	Outcome
BFl proximal	2.4 ± 3.0	22.4 ± 17.4**	1.44 ± 0.85	**↑**PLE
BFl medial	4.1 ± 3.5	11.2 ± 8.1**	1.05 ± 0.84	**↑**PLE
BFl distal	3.5 ± 3.2	5.0 ± 7.0	0.25 ± 0.84	Unclear
BFs proximal	17.8 ± 4.4**	4.7 ± 4.0	-2.95 ± 0.81	**↑**FLC
BFs medial	11.6 ± 3.2**	2.6 ± 4.8	-2.08 ± 0.82	**↑**FLC
BFs distal	8.7 ± 4.3**	1.9 ± 2.5	-1.80 ± 0.80	**↑**FLC
ST proximal	16.0 ± 4.2	12.8 ± 6.7	-0.53 ± 0.83	Unclear
ST medial	17.5 ± 4.0	21.3 ± 11.6	0.41 ± 0.84	Unclear
ST distal	14.7 ± 5.4**	3.1 ± 3.4	-2.41 ± 0.80	**↑**FLC
SM proximal	1.5 ± 1.6	7.1 ± 6.7*	1.03 ± 0.85	**↑**PLE
SM medial	1.5 ± 1.4	2.7 ± 5.3	0.27 ± 0.85	Unclear
SM distal	0.9 ± 1.1	5.2 ± 4.4**	1.23 ± 0.85	**↑**PLE

BFl, biceps femoris long head; BFs, biceps femoris short head; ST, semitendinosus; SM, semimembranosus.

**: Significantly different (p<0.01);

*: Significantly different (p<0.05).

Unclear correlations were obtained between the mechanical variables measured and changes in the T2 values of BFl, BFs, SM, and ST muscles (p>0.05).

## Discussion

The aim of the present study was to analyze mechanical responses and MRI-based regional muscle use during inertial knee- and hip-dominant hamstring strengthening exercises in professional soccer players. The main findings of the present study were: 1) CON mean power was always higher than ECC mean power in both exercises; 2) ECC overload was only observed in peak power during the VP; 3) VP (hip-extension exercise) allowed greater mean and peak power than LC (knee-flexion exercise), while players perceived a higher training load after LC exercise; 4) overall, changes in T2 values in most regions of the 4 muscle bellies, particularly the BFs and ST, were greater during the knee-flexion exercise (LC); and 5) VP appeared more capable of selectively engaging the BFl proximal and medial regions while all regions of BFs were selectively used by the LC.

Previous studies have shown a link between the mechanical stress applied to contractile tissue and muscle hypertrophy [43,44], and muscle stretch combined with overloading is one of the most effective stimuli for muscle growth [45]. Training session data from our study showed that for inexperienced professional soccer players there was no eccentric overload (defined as greater load during the ECC phase vs the CON phase) during LC exercise. While flywheel devices are a very convenient technology for emphasizing more forceful actions in the ECC-CON transition phase, it is unclear whether such devices can offer eccentric overload [31]. A previous study suggests that using the inertia provided by a rotating flywheel to provide resistance in leg-curl exercise may enable the production of eccentric overload [25]. Our results, in line with Nunez et al. [31], after analyzing all repetitions during the training session showed less mean (-29% to -35%, Table 1) and peak (-6% to -17%, Table 1) power during the ECC phase in comparison with the CON phase. Mainly, this was due to the substantial and excessive disparity in time between the phases (Fig 4), with time in ECC vs CON ratios increasing gradually as the sets were executed (from +42% to +61%). These findings are in line with or slightly higher than those of Tous-Fajardo et al. (2006), who found ECC vs CON ratios for mean power in experienced and inexperienced players of -54% to -65% and -44% to -65%, respectively, and ratios for peak power of -7% to -17% and -10% to -36% respectively. During the training session with VP, exercise data revealed less mean power during the ECC phase (-11% to -15%) while players reached higher peak power during the ECC phase (+22% to +15%), with lesser differences in time ECC vs CON ratios in comparison with LC exercise, which decreased gradually as the sets were executed. In addition to this, present results showed that CON and ECC mean power during the first set in VP was lower than in any other set. This was probably to the fact that the first set in VP with a 0.21964 kg/m^2^ moment of inertia in this population could be used as a protocol to stimulate the postactivation potentiation responses to obtain a subsequent better performance [46].

When prescribing hamstring strength training exercises, individual hamstring muscles are not activated in a similar manner [47] and changes in response to resistance training occur non-uniformly along the muscle [35,36]. The results of the present study showed that each individual hamstring muscle and regional zone responded differently during hip extension (VP) and knee flexion (LC) exercises. Previous studies showed that BFl and SM were selectively recruited during hip joint movements such as the “stiff-leg deadlift” exercise [7], whereas ST and BFs were mainly activated in knee flexion exercises such as the prone leg curl or Nordic Hamstring exercises [16,48], although there may also be certain activation in the middle and distal regions in BFl after the prone leg curl [16].

Our results indicated that in elite soccer players, T2 values after training using LC exercise were increased mainly in BFl, BFs and ST, as found in previous studies [14,16] and in contrast with a study that did not find changes in BFl after ECC LC [47]. In accordance with Mendez-Villanueva et al. [14], we used a flywheel Leg-Curl device with a high training load in the CON and ECC phases. That is probably why our results, like those obtained by Mendez-Villanueva et al. [14], showed greater recruitment in the proximal region in the BFl than was found by Kubota et al. [16], although generally the individual responses in BFl in our study showed substantially higher CV (41%–75%) than in ST or BFs, where more hamstring injuries occur and where the LC exercise has its greatest impact (7%-19%). Previous studies suggest that scar tissue formation in the muscle, along with weakness or atrophy of previously injured muscle, may be contributing factors to re-injury [49], and that after biceps femoris injuries, 85% of players likely return to sport with residual atrophy of the BFl and/or hypertrophy of the BFs [50]. This could lead to changes in the muscle-tendon unit and alter the contraction mechanics during functional movements (i.e. running), therefore contributing to re-injury risk [50]. It is not entirely clear why this atrophy in the BFl and/or hypertrophy of the BFs processes take place. There may be a possible compensatory effect by BFs after BFl injury and during the recovery process, which is enabled by the separate innervation of the long and short heads [48], or a recovery and/or return to sport process based mainly on strengthening the hamstring muscles through knee flexor exercises such as the Nordic Hamstring or leg curl [16,48]. The angle of the hip produces a greater impact on the length of the BFl than the angle of the knee [51,52], and as measured by MRI, hip flexion-extension exercises cause greater BFl recruitment compared with fixed-hip movements [7,14,53]. This is keeping with our results, in which VP hip flexion-extension exercise resulted in a selective and homogenous use of the proximal and medial regions of the BFl belly (Table 3). In contrast to Ono et al. [7] and in agreement with Mendiguichia et al. [48] for the lunge exercise, we didn’t find recruitment in the SM, since the mechanics of the hip flexion-extension exercises used were different from each other, possibly causing a different response. Physiotherapists and strength and conditioning coaches could consider this when optimizing the recovery process after BFl muscle injuries.

This study has limitations. The T2 changes reflect the metabolic response to muscle activation. In this regard, work done by tendons and/or other elastic structures reduces muscle work and therefore metabolic cost (i.e. T2), and can have a powerful effect on muscle power. This may explain why there were unclear correlations between power output and changes in T2 values. Although fMRI T2 change has been widely used for evaluating muscle recruitment during a wide range of exercises involving the hamstring muscles [14], this information needs to be interpreted with caution. A small sample size was used in the present study although with a population of elite athletes.

## Conclusions

VP hip-extension exercise produced greater mean and peak power than LC flywheel knee-flexion exercise, although players perceived a higher training load after LC exercise. The main benefits of both exercises were that LC exercise involved a greater overall use of the 4 muscle bellies, more specifically in the ST and BFs, with a selective augmented activity (compared with the VP) in the 3 regions of the BFs, while VP exercise selectively targeted the proximal and medial regions of the BFl, where more hamstring injuries occur [2,4,5]. These findings are of particular importance in prescribing a strengthening program for preventing hamstring injuries in soccer players. In addition, these findings could be crucial in reducing or eliminating residual atrophy of the BFl and hypertrophy of the BFs in previously injured muscle and probably minimizing the risk of re-injury [50]. The reason for this could be that in training with this exercise, the impact is mainly focused on the proximal and medial region of the atrophied BFl, with no influence on hypertrophied BFs. Future research is needed with experienced professional soccer players to identify longitudinal changes in mechanical variables and muscle volume after long-term training with each exercise.
